# Enhancement of rosmarinic acid biosynthesis in *Ocimum basilicum* L. via melatonin-loaded nanoparticles: insights into enzymatic activity and gene expression

**DOI:** 10.1186/s12870-026-08630-7

**Published:** 2026-09-02

**Authors:** Rabab Abdulhai Gad, Tahani Abbas Hathout, Samia Moheb El khallal, Ashraf Hussein Fahmy, Gihan Mohamed Hussein, Marwa Talat Elshamy

**Affiliations:** 1 Botany Department, Faculty of Women for Arts, Science and Education, Ain Shams University, Cairo, Egypt; 2 Agriculture Genetic Engineering Research Institute (AGERI), Agricultural Research Center, Giza, Egypt

**Keywords:** *Ocimum basilicum* L, Rosmarinic acid (RA), Melatonin nanoparticles (MEL NPs)

## Abstract

**Background:**

This study aimed to synthesize melatonin laoded chitosan nanoparticles (MEL NPs) and assess its effects on the production of bioactive metabolites, antioxidant activity and gene expression in *O. basilicum* culture.

**Results:**

Our results showed that the total phenolic (TPC) and total flavonoid (TFC) content of basil leaves treated with melatonin (MEL) and MEL NPs at 5, 10, and 20 μm was significantly increased, with the rosmarinic acid content also being enhanced. Treatment with 10 µM MEL NPs induced the most pronounced enhancement in total phenolic content (TPC) and total flavonoid content (TFC), reaching 3.48 and 3.79- fold, respectively, compared with the control. meanwhile, the same treatment resulted in the maximum accumulation of rosmarinic acid (RA), 19.73 mg g⁻¹ FW.

Furthermore, the application of MEL NPs or melatonin markedly stimulated the phenylprpanoid pathway in basil leaves, as evidenced by the enhanced activities of tyrosine aminotransferase (TAT) and phenylalanine ammonia-lyase (PAL). This enzymatic stimulation was accompanied by significant transcriptional activation of key pathway genes. Notably, basil leaves treated with 10 µM MEL NPs showed pronounced upregulation of 4-coumarate: CoA ligase (4*CL*) and Hydroxyphenylpyruvate reductase (*HPPR*) transcript with expression levels increasing by 3.45- and 5.47-fold, respectively, compared with the untreated control.

**Conclusion:**

The study concluded that nano encapsulated melatonin at 10 µM was the most effective elicitor for stimulating rosmarinic acid biosynthesis by coordinating transcriptional activation of key biosynthetic genes and enhancing tha activities of related enzymes in basil tissue culture. This is due to its stability and targeted delivery compared to conventional melatonin in large-scale *Ocimum basilicum* L. plant cultivation as a resource for food and pharmaceutical industries.

## Background

Medicinal plants produce myriad secondary metabolites; a review of Ruta graveolens catalogued more than 200 compounds, with phenylpropanoid coumarins and alkaloids dominating the nonvolatile fraction [1]. Genomic resources now link such metabolite diversity to biosynthetic genes: sequencing of the medicinal fungus Trametes sanguinea generated a 41.3 Mb genome with ~ 10,886 annotated genes and highlighted pathways for its antitumor and antimicrobial polysaccharides [2]. In plants, a genome wide analysis of WUSCHEL related homeobox genes in ramie identified 17 members and associated elevated BnWOX10 and BnWOX14 expression with adventitious root formation, underscoring the importance of regulatory genes in vegetative propagation [3]. Meanwhile, atmospheric and room temperature plasma mutagenesis coupled with whole genome resequencing has emerged as a rapid way to identify functional genes; in Pseudomonas fluorescens this approach pinpointed three GGDEF EAL genes whose deletion elevated c di GMP and biofilm formation [4]. Such advancements illustrate how the integration of phytochemical, genomic, and mutagenesis tools can effectively inform targeted metabolic engineering. Based on this rationale, our study evaluates the capacity of nano-encapsulated melatonin to modulate key enzymes and gene expression within the phenylpropanoid pathway, thereby enhancing rosmarinic acid accumulation in *O. basilicum*.

Rosmarinic acid (RA), an ester derivative of caffeic acid and 3,4-dihydroxyphenyllactic acid, is predominantly found in numerous species within the Lamiaceae and Boraginaceae families. Consequently, it is also present in various medicinal herbs and culinary spices [5]. It is a major phenolic compound in the basil tissues [6]. RA exhibits a broad spectrum of bioactivities, including antimicrobial, antioxidant, antiviral, and anti-inflammatory effects [5]. Recent research has shown that RA can prevent SARS-CoV-2 from entering the body and replicating [7]. The enzymes phenylalanine ammonia-lyase (PAL) and tyrosine aminotransferase (TAT) are essential to produce rosmarinic acid (RA) in the Lamiaceae family, which involves two amino acid precursors, L-phenylalanine ammonia-lyase and L-tyrosine [8]. There were 22 genes linked to RA biosynthesis that have been found by transcriptome analysis, including 4*CL* and *HPPR* [9].

Developing in vitro cultivation methods as a viable substitute to meet the demand for medicinal plants has been the focus of current developments [10]. Therefore, there is a paradigm change in favor of using plant tissue culture techniques to generate important secondary metabolites in vitro [11]. In this regard, melatonin-loaded chitosan nanoparticles have emerged as a novel and potent abiotic elicitor.

Bioactive substances obtained from natural plants have attracted increasing attention in recent years, especially antioxidant molecules like melatonin (MEL), also known as N-acetyl-5-methoxytryptamine, which was first discovered in plants in 1995 [12]. Since then, it has been recognized as a pleiotropic molecule that contributes to several physiological processes, including plant growth, development, and defense against biotic and abiotic stressors [13]. Apart from plants, melatonin has a variety of biological functions that could support human health maintenance [14]. Recent research has shown that melatonin (MEL) regulates secondary metabolism by encouraging the buildup of phenolic substances, such as anthocyanins, carotenoids, total phenols, and flavonoids [15–18]. Additionally, MEL promotes the production of rosmarinic acid (RA) by boosting the activity of RA-related enzymes and upregulating related gene expression [16, 19]. Furthermore, MEL raises levels of soluble sugar, especially sucrose and sorbitol and modulates primary metabolism. In addition to its metabolic effects, MEL reduces oxidative stress by scavenging hydrogen peroxide (H₂O₂) and boosting the activity of antioxidant enzymes [18–22]. However, the application of traditional chemical elicitors remains constrained by suboptimal bioavailability, inherent instability, and the necessity for recurrent treatments, which often yield only transient metabolic responses [23]. These limitations underscore the urgent need for innovative elicitation strategies that ensure sustained and high-yield metabolite production while maintaining cost-effectiveness and environmental sustainability [24].

Nanoparticles (NPs) have been used as an innovative method for encouraging the synthesis of bioactive compounds in plants. Constructing, modifying, and using materials at the nanoscale (1–100 nm), where they have special physical, chemical, and biological characteristics different from those of their bulk counterparts, is known as nanotechnology [25]. Because of these characteristics, NPs have demonstrated potential uses in a variety of industries, such as biotechnology, electronics, food, medicine, and agriculture [26]. The role of NPs as elicitors has been extensively investigated by recent studies, which have demonstrated their effectiveness in enhancing the synthesis of bioactive chemicals in plants and plant cell cultures under various culture conditions [27–29]. The most significant advantage of nanoparticle-mediated elicitation is the ability to deliver bioactive molecules directly to the plant’s metabolic machinery [30]. This targeted approach circumvents the limitations associated with conventional elicitors in cell cultures, such as rapid degradation, heterogeneous distribution, and transient exposure—factors that frequently induce cellular stress and result in suboptimal metabolite accumulation [31]. Furthermore, abiotic elicitors, including nanoparticles, modulate the production of specialized metabolites by stimulating the transcription of key genes within defense-related biosynthetic pathways [32].

Plant hormone-loaded nanoparticles (NPs) represents a class of biotechnological tools, offering substantial potential for plant cell culture due to their unique physicochemical properties [33]. Consequently, the present study focuses on the synthesis of melatonin-loaded chitosan nanoparticles (MEL NPs) and evaluates their impact on the metabolic and physiological profiles of *Ocimum basilicum* cell cultures. Chitosan, a biocompatible, biodegradable, and GRAS-approved polysaccharide, is widely used in drug delivery due to its ability to control the release of active compounds [34]. The ionic gelation technique, based on electrostatic interactions between chitosan and anionic crosslinkers, is a preferred method for preparing CS nanoparticles due to their safety, simplicity, and solvent-free nature [35]. Melatonin therapy increased the transcription levels of genes involved in anthocyanin biosynthesis [36]. *Ocimum basilicum* L. callus generated the monoterpene hydrocarbons eugenol, oxygenated monoterpenes linalool, and hydrocarbons and derivatives tetradecane and p-allylanisole and rosmarinic acid grown on a 100 µM melatonin medium [6]. Vase solutions supplemented with encapsulated melatonin in nanochitosan (nCS-Mel) at concentrations of 0.1 and 0.5 mM significantly enhance the postharvest quality of cut *Gerbera jamesonii* flowers. Compared to conventional melatonin treatments, the nCS-Mel formulation promotes higher anthocyanin accumulation, maintains superior cell membrane integrity, and elevates the levels of total carbohydrates and flavonoids within the petals [37]. The jasmonic acid (JA) signaling pathway depends on the NPs’ ability to efficiently transfer JA into plant cells. This route controls important enzymes involved in the manufacture of SMs, including terpenoids, flavonoids, and alkaloids [38]. Utilizing JA and interacting with Fe_3_O_4_ NPs also improved the uptake of nutrients and secondary metabolite, which has a favorable impact on cell development and metabolism in general [39, 40]. Previous investigation study has demonstrated that the induction of secondary metabolites, Glycyrrhizin, total phenolic compounds, and anthocyanins in Glycyrrhiza glabra plant seedlings is positively impacted by nanoparticles such as ZnO and CuO Nps compared to the bulk size ZnO and CuO particles [41]. The production of artemisinin was enhanced by the stimulation of silver nanoparticles [42]. Impact of foliar spraying with melatonin and chitosan Nano-encapsulated melatonin on tomato (*Lycopersicon esculentum L*. cv. Falcato) plants under salinity stress effectively alleviate salinity-induced stress, encouraging growth and preserving physiological homeostasis [43].

The objective of this investigation is to synthesize chitosan (CS)-based nanoparticles as a robust delivery platform for melatonin to enhance its stability, bioavailability, and elicitation efficiency. This study evaluates the efficacy of these nano-formulations in stimulating the biosynthesis of rosmarinic acid, total phenolic and flavonoid contents, and the activities of key enzymes (PAL and TAT). Furthermore, the research assesses the transcriptional upregulation of pivotal biosynthetic genes (4*CL* and *HPPR*) in *Ocimum basilicum* under in vitro culture conditions, providing a comparative analysis between nano-encapsulated melatonin and its conventional counterpart.

## Materials and methods

Chitosan (C_6_H_11_NO_4_)_n_ (MW 250 KDa, degree of deacetylation 97%) and sodium tripolyphosphate (TPP) were acquired from St. Louis, Missouri-based Sigma-Aldrich. Melatonin (C_13_H_16_N_2_O_2_) was purchased from a science lab (Texas, USA). Basil (*Ocimum basilicum* L.) seeds were supplied by the National Gene Bank of Egypt (NGB) (Landrace L10 cultivar), Agriculture Research Center, Ministry of Agriculture, Giza, Egypt.

### Preparation MEL nanoparticles

Polymeric nanoparticles of MEL were prepared using the ionic gelation method according to [44] with some modifications [45, 46]. First, the 1% CS (1 mg ml^− 1^) was dissolved in 1% glacial acetic acid, on PH 5.5. Melatonin (2.32 mg) was added in 100 ml chitosan solution to a concentration of 100 µM l^− 1^ and allowed an hour to interact. Afterward, sodium tripolyphosphate (0.5 mg/mLTPP) was used to create nanoparticles by cross-linking. The ratio of chitosan solution and TPP was optimized to be 3:1 v/v. The pH was adjusted to 5.7 after adding TPP aqueous solution dropwise. The reaction was continuously stirred (800 rpm) and kept at room temperature in the dark overnight. Opalescent dispersion is achieved, which indicates the formation of nanoparticles. Loaded NPs were gathered by centrifugation for 30 min at 10,000 rpm at 4 °C, and the precipitate was dried in an oven at 60 °C for 12 h to obtain a reddish-brown powder of melatonin nanoparticles.

### Characterization of MEL nanoparticles

#### The encapsulation efficiency (EE)

Encapsulation efficacy (EE) of melatonin encapsulation on chitosan nanoparticles was determined according to [45] with some modification as reported by [47]. The absorbance was measured by a UV spectrophotometer for the water phase, which contains un-encapsulated melatonin at 278 nm. The Free Melatonin in Supernatant concentration was calculated according to the the melatonin standard curve (1 mg/ml as stok solution) at concentrations (20, 60, 80, 100 µM). The encapsulation efficiency (EE) was calculated by the equations as follows:

$$\:\mathrm{E}\mathrm{E}\mathrm{\%}\:=\:\frac{(\mathrm{T}\:\mathrm{M}\mathrm{e}\mathrm{l}\mathrm{a}\mathrm{t}\mathrm{o}\mathrm{n}\mathrm{i}\mathrm{n}-\:\mathrm{S}\:\mathrm{M}\mathrm{e}\mathrm{l}\mathrm{a}\mathrm{t}\mathrm{o}\mathrm{n}\mathrm{i}\mathrm{n})}{\mathrm{T}\:\mathrm{M}\mathrm{e}\mathrm{l}\mathrm{a}\mathrm{t}\mathrm{o}\mathrm{n}\mathrm{i}\mathrm{n}\:}\times\:\:100\mathrm{\%}$$

In this case, EE stands for encapsulation efficiency, T for total melatonin content, and S for free MEL content in the supernatant.

#### Dynamic light scattering (DLS)

Dynamic Light Scattering (DLS) was used to determine the average hydrodynamic particle size, polydispersity index (PDI), and zeta potential of MEL nanoparticles using a Malvern Zetasizer (Malvern Instruments, UK) at a wavelength of 532 nm at 24.9^◦^ C with a detection angle of 90° according to [48]. Prior to analysis, the nanoparticles were diluted properly (1:10, v/v) with deionised water to prevent multiple scattering effects, sonicated for 10 to 15 min to reduce aggregation, The calculations were performed using refractive index values of 1.33 for the dispersant water.

#### Transmission electron microscopy (TEM)

According to [49], particle size and shape of MEL nanoparticles were detected by TEM analysis, a drop of the solution was placed on the carbon coated copper grids (CCG) and dried by allowing water to evaporate at room temperature. Electron micrographs were obtained using JEOL JEM-1010 transmission electron microscope at 80 kV at. To ascertain the mean particle size and size distribution, six individual nanoparticles were analyzed from the figure.

#### Fourier-transform infrared spectroscopy analysis (FTIR)

FTIR analysis was performed to determine the functional groups and potential chemical interactions involved in the creation of MEL nanoparticle according to [50]. Measuring liquid samples with Attenuated Total Reflectance (ATR) FTIR Bruker model Alpha II involves placing a small drop of liquid sample directly onto a cleaned Diamond crystal to form a thin evanescent layer and the sample is then cleaned off with a suitable solvent. The results were recorded by ALPHA II (Bruker Optik GmbH, Germany) and the data were recorded in the range of 4000 to 400 wave cm^− 1^.

### Growth conditions and treatments

Seeds from NGB, for 10 min, were surface sterilized using a 30% (v/v) solution of commercial bleach (Clorox^®^, The Clorox Company; 5.4% sodium hypochlorite) [51], rinsed three times with sterile distilled water for 3 min. Subsequently, in a laminar flow hood. Seven sterilized basil seeds were cultured onto 50% Murashige and Skoog (MS) basal medium components as shown in Table 1, with 3% sucrose (w/v) and solidified with 8.1 g l^− 1^ agar without any hormone. The PH was adjusted to 5.8 using 1 N KOH or 1 N HCL. The experiment was arranged in a completely randomized design (CRD), with thirty glass plant tissue culture jars (8 cm in diameter × 15 cm in depth; 12 oz [350 mL]) per treatment. Equal amounts of the medium (50 ml/jar) were put into culture jars and sealed with polypropylene cap and then autoclaved for 20 min at 121 °C and 1.1 kg cm^− 2^ pressure [52].

**Table 1:** Murashige and Skoog medium composition

Ingredients	Amount (mg/l-1)
Macronutrients	
NH_4_NO_3_	1650.00
KNO_3_	1900.00
CaCl_2_.2H_2_O	440.00
MgSO_4_.7H_2_O	370.00
KH_2_PO_4_	170.00
Micronutrients	
KI	0.83
H_3_BO_3_	6.20
MnSO_4_.4H_2_O	22.30
ZnSO_4_.7H_2_O	8.60
Na_2_MoO_4_.2H_2_O	0.25
CuSO_4_.5H_2_O	0.025
CoCl_2_	0.025
Iron stock	
FeSO_4_.7H_2_O	27.80
Na_2_.EDTA.2H_2_O	37.30
Vitamins	
Myo-inositol	100.00
Nicotinic acid	1.00
Pyridoxine HCl	1.00
Thiamine HCl	10.00
Glycine	2.00

Sterilized seeds were inoculated per jar. Cultures were incubated under a 16/8 (light/dark) photoperiod of cool-white, fluorescent light (80 µmole m^− 2^ s^− 1^) at 25 ± 2 °C until full germination. The experiment was conducted using (sweet basil plant), and the media were treated according to a randomized complete block design with six treatments:, (1) Untreated control; (2) Normal MEL (100 µM) (Treated control); (3) 5 µM MEL NPs; (4) 10 µM MEL NPs; (5) 20 µM ME NPs; (6) 30 µM MEL NPs. Seven seeds of *O. basilicum* were planted on August 15 and harvested on September 14. The plants were removed from the media and used to measure the change in physiological, biochemical, and genetic studies.

### Extraction and estimation of total phenolic content (TPC)

The total phenolic content (TPC) was assayed by grinding about 0.3 g leaf samples in liquid nitrogen and extracted with 5 mL of 80% ethanol. The methanolic extracts were then Shaked by shaker at 37 °C and 100 rpm overnight. Then, the extracts were filtered prior to further analysis.

The Folin-Ciocalteu reagent was used to determine the total phenolic content as described by [53]. 1 ml of the sample extract was mixed with 5 ml of the Folin-Ciocalteu reagent (2 N). Then, after 5 min, 4 ml of 7.5% (w/v) sodium carbonate (Na_2_CO_3_) was added. After incubation at room temperature for 2 h in darkness, the absorbance of the reaction mixture was measured at 740 nm using a spectrophotometer. TPC was calculated from a gallic acid standard curve **(**0.1, 0.2, 0.3, 0.4, 0.5 and 0.6 mg/mL**)** and expressed as mg gallic acid equivalents g⁻¹ FW.

### Extraction and estimation of total flavonoid content (TFC)

To extract the total flavonoid content, 0.3 g of leaves were homogenized in liquid nitrogen within 5 ml of 80% ethanol. The extract was kept overnight in a shaker set at 37° and 100 rpm and then the extracts were filtered. An aluminum chloride (AlCl_3_) reagent was used to determine the total flavonoid levels [54]. Ethanolic sample extract (0.5 ml) was mixed with 100 µl of 10% (w/v) aluminum nitrate, 100 µl of potassium acetate (1 M), and 4.3 ml of 80% ethanol to achieve a total volume of 5 ml and incubated at room temperature for 40 min. The absorbance was determined at 415 nm after incubation. Quercetin was used as a standard for constructing the calibration curve with concentrations of (0.2, 0.4, 0.6, 0.8, and 1.0 mg/ml). The flavonoid content was calculated using the calibration plot and expressed as mg quercetin equivalents g^− 1^ FW.

### Determination of rosmarinic acid content by high-performance liquid chromatography (HPLC)

High-performance liquid chromatography (HPLC) was used to quantify the RA, *O. basilicum* samples were frozen in liquid N2 and pulverized with a mortar and pestle to fine powder. The extraction was carried out utilizing the guidelines provided by [55] with some modifications as [56]. RA extraction was taken from samples (0.5 g) with 1.25 mL of 0.1% acetic acid and 5 ml of 80% methanol (MeOH) mixture in 1:4 ratio. Noteworthy, extraction twice is ideal for analysis.The extracted samples were continuously stirred at room temperature for 24 h and then centrifuged for 5 min at ambient temperature at 12,000 rpm. After filtering and vacuum drying to dryness, they were dissolved in one ml of methanol, before analysis by HPLC.

The RA solution was run through the Agilent Series 1100 HPLC equipment (Agilent, USA) that included two LC- pumps (series 1100), a solvent degasser, an auto-sampling injector, and ChemStation software., UV/Vis’s detector (set at 250 nm). and a C18 reverse-phase column (125 mm × 4.60 mm; 5 μm particle size RStech, Daejeon, Korea). The analysis was completed at room temperature, and a UV detector set to 250 nm was used for detection. The conditions of HPLC and gradient program followed the protocol described by [39] with some modifications. In the mobile phase, solvents A and B were 4% acetic acid in water and 100% methanol, respectively. 60% solvent A and 40% solvent B made up the initial proportion of the mixture. After 10 min, the solvent gradient reached 50% solvent B, and after another 5 min, it reached 60% solvent B. After that, it stayed at 60% solvent B for 10 min. The solvent flow rate was 0.8 ml min^− 1^, and the injection volume was 25 µl. Standard solution of rosmarinic acid was prepared by dissolving the standard in HPLC grade methanol to produce stock solution of 2 mg/ ml. Results were expressed as mg RA g⁻¹ FW.

### Assay of certain enzymes involved in rosmarinic acid biosynthesis

#### Assay of Tyrosine aminotransferase enzymes activity (TAT; EC 2.6.1.5)

The activity of TAT enzyme was measured based on the production of p-coumaric acid from L-tyrosine. Firstly, 0.2 g of the frozen leaf samples was homogenized in 2 ml of 0.1 M phosphate buffer (Na_2_HPO₄ and NaH_2_PO₄) (pH 8.0) containing 1 mM dithiothreitol (DTT) on ice. The extracts were centrifuged at 12,000×g at 4 °C for 20 min and the supernatant was used for assay of TAT activity.

Activity of TAT was estimated based on the methodology of [16]. After extracting 0.1 ml of the supernatant was mixed with 0.1 M phosphate buffer (pH 8.8). The substrate 4 mM L-tyrosine (5.5 µM) was added to start reaction followed by incubation at 37 °C for 1 h. To stop the reaction, 0.05 ml of HCl (6 M) was added. At 330 nm, the absorbance of the sample was measured against the blank (all mixture solutions without enzyme extract) using a spectrophotometer. TAT activity was expressed in (U g^− 1^). One unit will be producing 1 µmole of p-coumaric acid through the biotransformation of L-tyrosine with a liberate of ammonia per minute at pH 8.8 at 37 °C.

#### Assay of Phenylalanine ammonia-lyase activity (PAL; EC 4.3.1.5)

The fine powder (0.5 g) was homogenized in extraction buffer 50 mM Tris-HCl (pH 8.8) containing 1 mM ethylenediaminetetraacetic acid (EDTA) ,10 mM 2-β-mercaptoethanol, and 2.5% polyvinylpyrrolidone-40 (PVP) in a 1:2 proportion (sample weight determined on a fresh weight basis/buffer). The mixture was centrifuged at 10,000 rpm for 20 min at 4 °C. An aliquot of supernatant was used as a crude enzyme extract.

Activity of PAL was estimated according to the method of [57]. 400 µl of 100 mM Tris-HCl (pH 8.8) buffer and 200 µl of 20 mM L-phenylalanine a crude enzyme extract was combined with 200 µl of the sample’s supernatant. After 30 min at 37 °C, 200 µL of 4 M HCl was added to stop the reaction. The change in color was measured at 290 nm using a spectrophotometer was measured against the blank (all mixture solutions without enzyme extract), to determine the quantity of trans-cinnamic acid that produced. PAL activity was expressed in (U g^− 1^). One unit will deaminate 1 µmole of L-phenylalanine to trans-cinnamate per minute at pH 8.8 at 37 °C.

### Quantitative real-time PCR analysis of rosmarinic acid biosynthesis genes in *O. bassilicum*

The transcriptional expression of rosmarinic acid biosynthetic genes, namely 4-coumarate: CoA ligase (4*CL*) and hydroxyphenylpyruvate reductase (*HPPR*) were detected in Ocimum basilicum leaves at 15- and 30-days post-inoculation using quantitative real-time PCR (qRT-PCR). Total RNA was extracted from leaf tissues using the TRIzol method [58]. RNA and cDNA synthesis was performed using a commercial kit (SolGent Co., South Korea) and oligo(dT)18 primers in accordance with the manufacturer’s guidelines.

qRT-PCR reactions were prepared using SolGent-Solg™ฏ M 2X Real-Time PCR Smart Mix containing SYBR^®^ฏ Green. Each 10 µL reaction comprised 5 µL of 1X master mix, 1 µL of 1:5 diluted cDNA template (from untreated and treated samples at various time points), 0.4 µL of each forward and reverse primer (10 µM), and nuclease-free water to volume. Primers for target genes and the endogenous reference gene (ubiquitin) are listed in Table 2. The selected reference gene (GenBank accession LN999820.1) has been previously validated for expression stability in the same accession background under comparable experimental conditions [59]. Cycle conditions included a 20-s initial denaturation at 95 °C, 40 cycles of 95 °C for 10 s, and *Ubiquitin* annealing/extension at 60 °C for 4*CL* and *HPPR* for 1 min. No-template controls (NTCs) were included to verify reaction specificity. Three independent biological replicates and each sample was analysed in technical duplicate in the qPCR analysis and relative gene expression values were calculated using the 2^-ΔΔCT method [60]. All experimental design parameters and methodological details are reported in line with MIQE recommendations to ensure transparency and reproducibility.

**Table 2:** List of primer sequences specific to *Ocimum basilicum* L. that are used in qRT-PCR (quantitative real-time PCR) tests

Target Genes	Primer Sequence (5’ to 3’)	Amplicon size (bp)	Accession No.
*Ubiquitin*	F: 5′- CATCACATTGGAGGTGGAGAG − 3′ R: 5′- TCCCAGCAAAGATCAACCTC − 3′	107	LN999820.1
*HPPR*	F: 5′- GCAGAATTGGGTTAGCAGTTG − 3′ R: 5′- CGCTGTCGTAGTACTTGTAGTC − 3′	108	KJ004761.1
*4CL*	F: 5′- CAACGCCTCCAGATCCAAAT − 3′ R: 5′- CGATGGTAACGACGGAGAAATC − 3′	113	KC576841.1

Data were statistically analyzed by SPSS v20.0 (Chicago, IL, USA). The statistical analysis was performed using one-way analysis of variance (ANOVA) and the significant differences between means were compared using Duncan’s multiple range test at *P* < 0.05.

### Statistical analysis

All experiments were carried out in triplicate to ensure the reproducibility and reliability of the data. Three independent biological replicates obtained from separate plant samples were used to assess biochemical parameters; each replicate was taken from an independent jar. For gene expression analyses (qRT-PCR) technical duplicates of each biological sample were used.

All the data was subjected to one-way statically analysis of variance (ANOVA) according to [61] using the SPSS software (SPSS version 20, USA). Different letters above the bars denote statistically significant differences between treatments according to Duncan’s multiple range test (*P* < 0.05).

## Results

### Characterization of nanoparticle

**Fig. 1:**
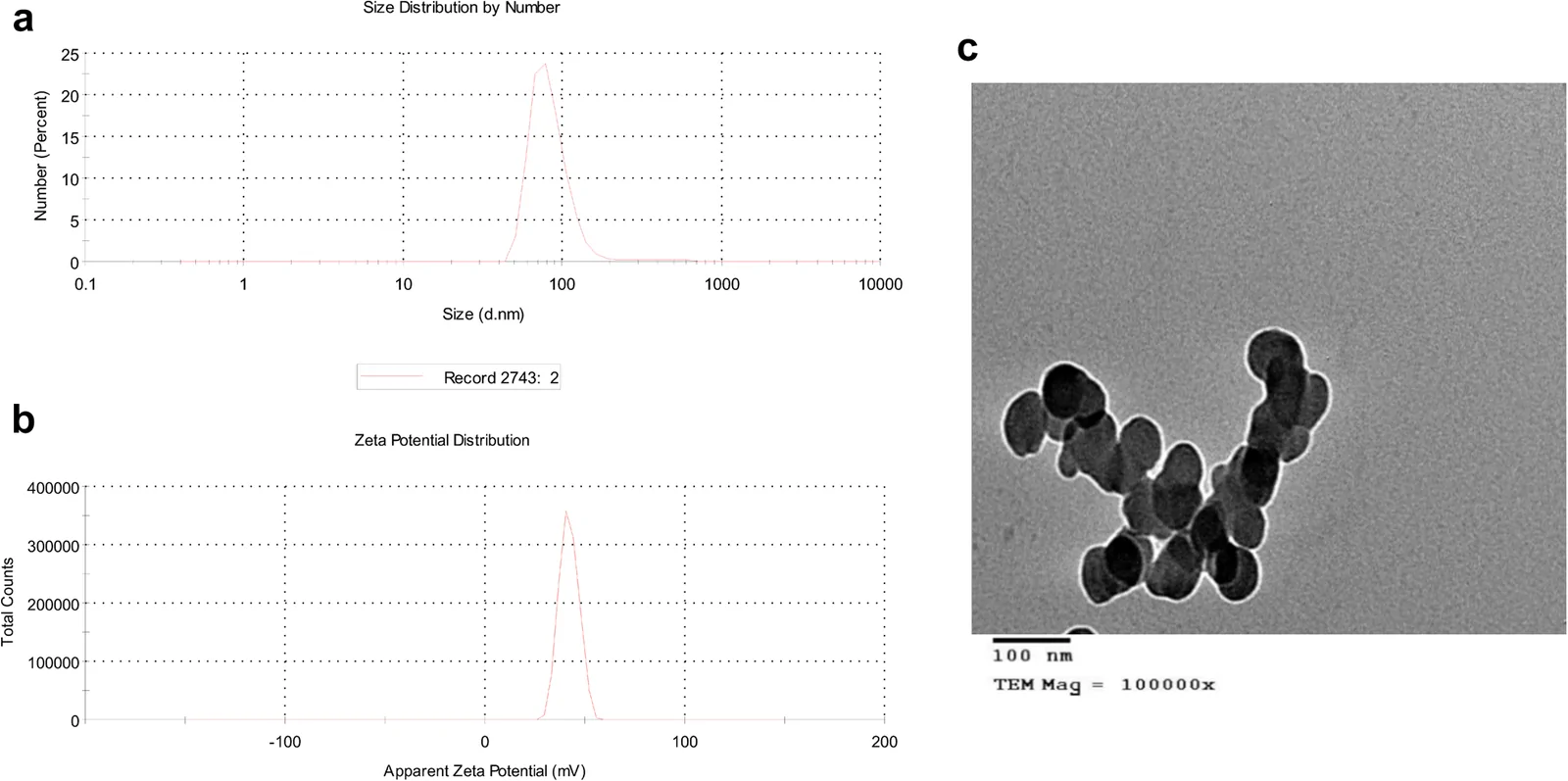
The size distribution by number and mean diameter (**A**), zeta potential distribution (**B**), and transmission electron microscopy images (**C**) for MEL nanoparticles

The encapsulation efficiency (EE%) for 100 µM melatonin within the chitosan nanoparticles was determined to be 78%. Physical characterization of the resulting MEL nanoparticles by using DLS (Fig. 1A; Table 3) revealed a highly uniform population with a single distinct peak, a number distribution of 98.8% and a mean diameter of 83.46 ± 0.634 nm (*n* = 3). Zeta potential (Fig. 1B) was recorded at + 42 mV, indicating high colloidal stability due to significant electrostatic repulsion between the positively charged particles. Morphological analysis via TEM (Fig. 1C) confirmed spherical nanoparticles with a smooth surface and a mean diameter of 74.61 ± 1.4 nm) of six particles were analyzed showing a uniform size distribution consistent with the DLS data.

**Table 3:** Characterization parameters of melatonin nanoparticles (MEL NPs)

Parameter	Value
Encapsulation Efficiency (EE%)	78%
Number Distribution	98.8%
Mean Diameter	83.46 ± 0.634 nm
Zeta Potential	+ 42 mV
Mean Diameter via TEM	74.61 ± 1.4 nm

**Fig. 2:**
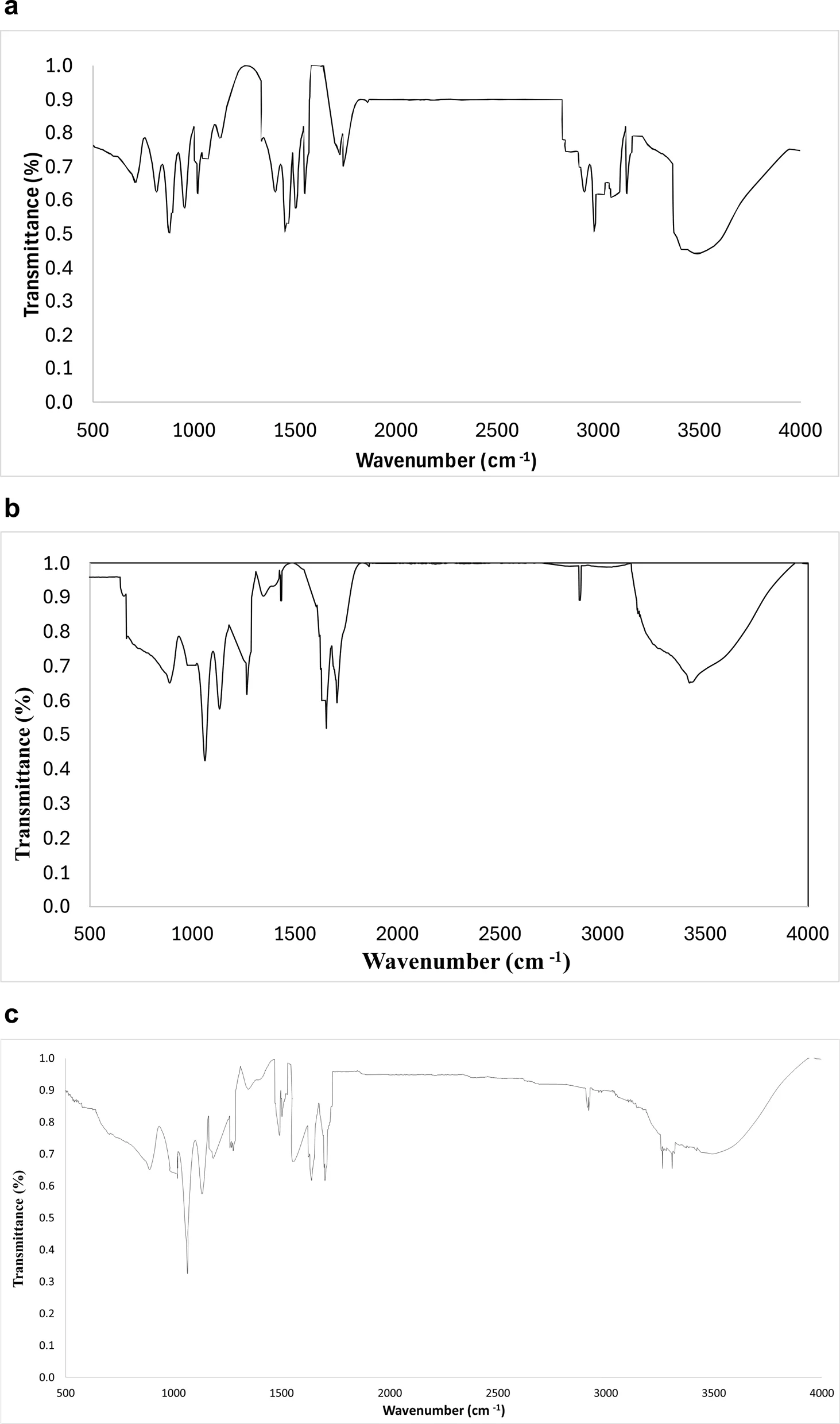
FTIR absorption spectra of MEL (**A**), CS (**B**), and MEL NPs (**C**)

The FTIR spectra for MEL, CS, and MEL NPs are illustrated in Fig. 2. Analysis of the MEL spectrum (Fig. 2A; Table 4) revealed characteristic peaks at 1180, 1680, 3100, 3260, and 3306 cm⁻¹, alongside two prominent sharp peaks at 1492 and 1550 cm⁻¹. The CS spectrum (Fig. 2B; Table 4) displayed primary absorption bands at 1080, 1370, and 1580 cm⁻¹, with a narrow peak at 2870 cm⁻¹ and a broad band spanning 3360–3450 cm⁻¹. For the synthesized MEL NPs (Fig. 2C; Table 4), new absorption bands emerged at 888, 1130, and 1210 cm⁻¹. Significant modifications in absorption intensity were recorded at 1530 and 1650 cm⁻¹, accompanied by a distinct spectral shift in the 3100–3450 cm⁻¹ region, indicating successful interaction and nanoparticle stabilization.

**Table 4:** FTIR spectral peak assignments for MEL, CS, and MEL NPs

Sample	Peak Position wavenumber (cm⁻¹)	Assignment
MEL	3306, 3260, 3100	N–H stretching (primary/secondary amines)
	1680	C = O stretching (Amide I)
	1550, 1492	N–H bending / C–N stretching
	1180	C–O–C stretching
CS	3450–3360	O–H and N–H stretching (broad)
	2870	C–H stretching
	1580	N–H bending (amine group)
	1370	C–N stretching
	1080	C–O–C stretching (pyranose ring)
MEL NPs	1650, 1530	Shifted Amide I & II (interaction peaks)
	1210, 1130, 888	New fingerprint regions / Cross-linking
	3450–3100	Broadened/Shifted H-bonding network

### Total phenolic and total flavoniod content

**Fig. 3:**
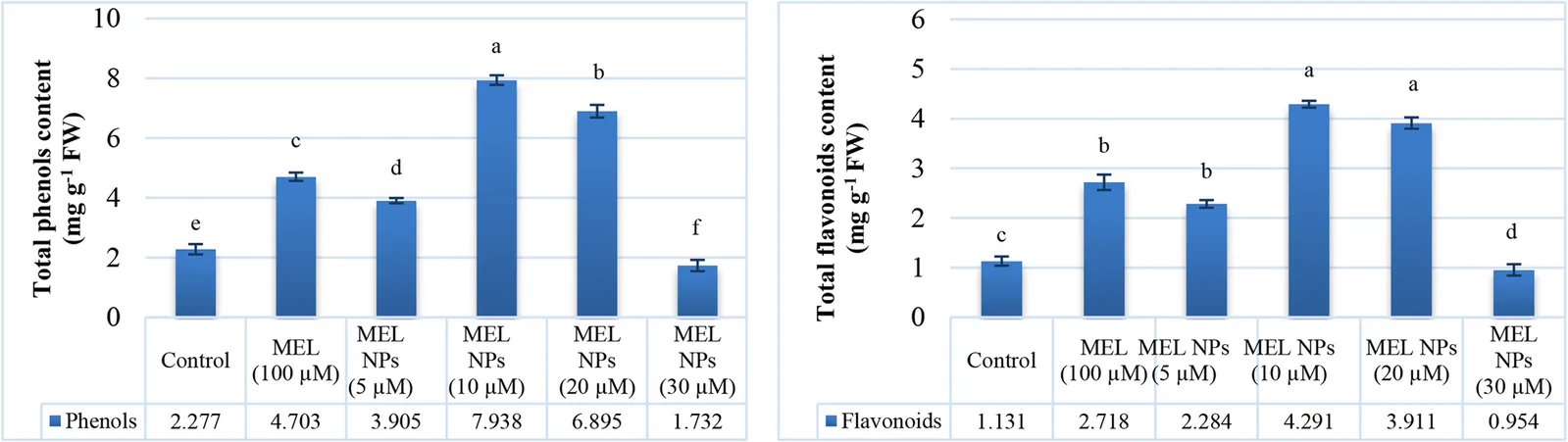
Total Phenolic Content (TPC) and Total Flavonoid Content (TFC) of *Ocimum basilicum* L. leaves 30 days post-melatonin treatments (control, conventional melatonin, and MEL NPs at various concentrations). Data points represent mean of three independent replicates (*n* = 3), with vertical bars indicating the standard deviation (± SD) using one-way ANOVA test. Different letters above the bars denote statistically significant differences between treatments according to Duncan’s multiple range test (*P* < 0.05)

The results of the analysis of variance (ANOVA) for secondary metabolite profiles, TPC, TFC, and RA content showed statistically significant variation between treatments, indicating the strong impact of the applied treatments on plant metabolic responses. The untreated control basil plants were utilized to establish baseline metabolite levels where recorded TPC of 2.277 ± 0.93 mg g⁻¹ FW and TFC of 1.131 ± 0.171 mg g⁻¹ FW (Fig. 3). All treatments significantly induced these metabolites, except the highest nanoparticle dose 30 µM MEL NPs group, which yielded the lowest values 1.732 mg g⁻¹ FW of TPC and 0.954 mg g⁻¹ FW ofTFC, which represent 0.76 and 0.844 Fold decreases, respectively. MEL at 100 µM increased TPC and TFC to 4.70 ± 0.156 and 2.72 ± 0.161 mg g⁻¹ FW, representing 2.06- and 2.41-fold increases over the control, respectively. Notably, 10 µM MEL NPs treatment resulted in the most significant enhancement at *p* < 0.05, where TPC reached 7.94 ± 0.114 mg g⁻¹ FW (3.48-fold) and TFC reached 4.29 ± 0.211 mg g⁻¹ FW (3.80-fold) compared with the control. Compared to conventional melatonin, the 10 µM MEL NPs treatment further elevated TPC and TFC by 1.68-fold and 1.57-fold, respectively.

### Rosmarinic acid content

**Fig. 4:**
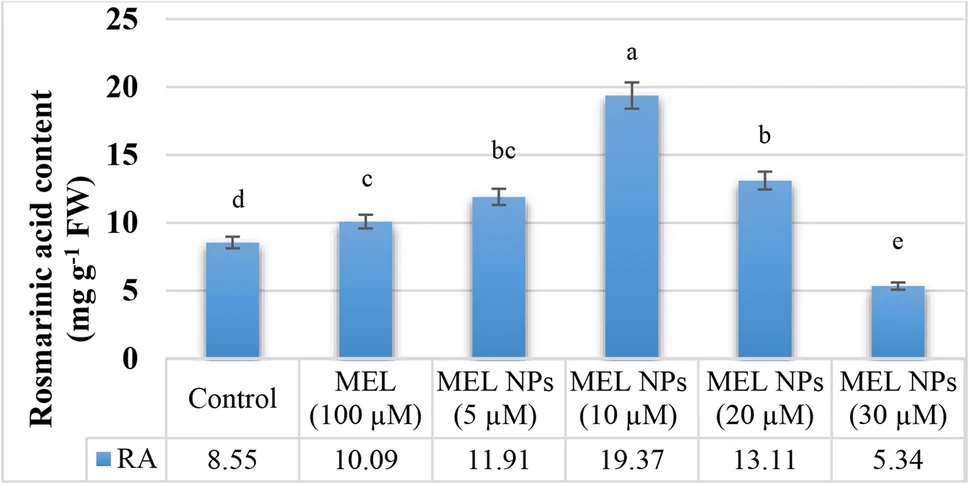
Rosmarinic acid (RA) content in *Ocimum basilicum* L. leaves 30 days post-treatment (control, conventional melatonin, and MEL NPs at different concentrations). Values represent mean of three independent replicates (*n* = 3), with vertical bars indicating the standard deviation (± SD) using one-way ANOVA test. Different letters above the bars denote statistically significant differences between treatments according to Duncan’s multiple range test (*P* < 0.05)

Regarding RA content, the control Basil plants was 8.55 mg g⁻¹ FW (Fig. 4). The production of RA was significantly impacted by the dose-dependent application of MEL NPs. In contrast to the standard 100 µM MEL treatment, which only reached 10.09 mg g⁻¹ FW (a 1.18-fold change), low-dose MEL NPs at 5 µM achieved a RA content of 11.91 mg g⁻¹ FW (a 1.39-fold change) compared to untreated control. Treatment with 10 µM MEL NPs significantly increased (*p* < 0.05) RA accumulation to 19.37 mg g⁻¹ FW (Fig. 4), representing a 2.27-fold increase over the control and a 1.91-fold increase compared to conventional melatonin (10.09 mg g⁻¹ FW). A significant inhibitory impact was observed at 30 µM MEL NPs, where RA content decreased to 5.34 mg g⁻¹ FW relative to the control.

### Tyrosine aminotransferase and Phenylalanine ammonia-lyase enzymes activity

**Fig. 5:**
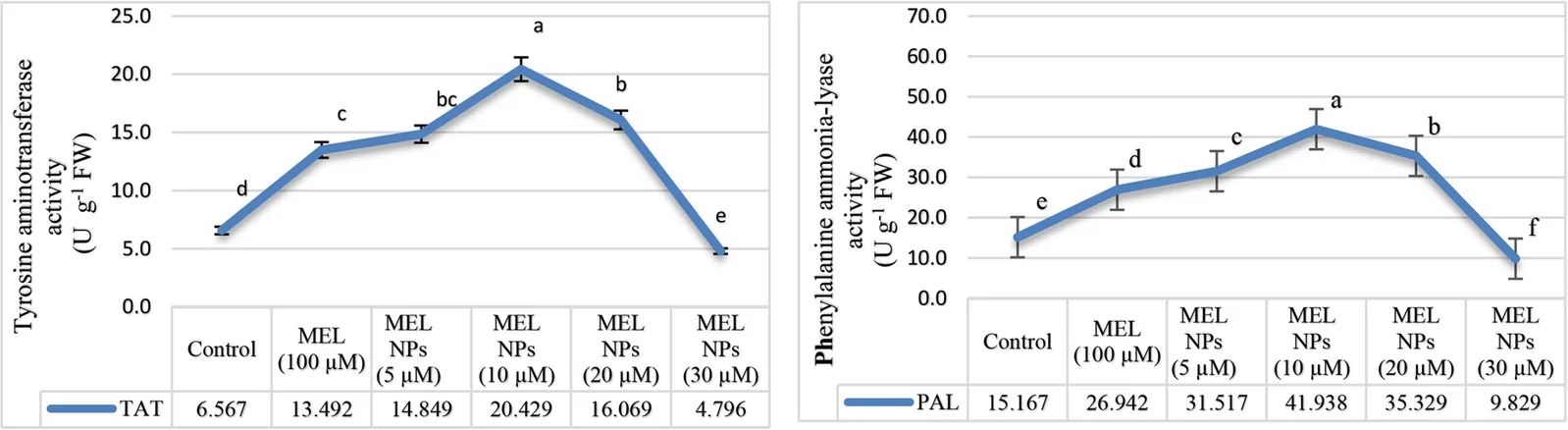
Influence of various melatonin treatments on TAT and PAL enzymatic activities in *Ocimum basilicum* L. leaves 30 days post-treatmet. Values represent the mean of three independent replicates (*n* = 3), with vertical bars indicating the standard deviation (± SD) using one-way ANOVA test. Different letters above the bars denote statistically significant differences between treatments according to Duncan’s multiple range test *P* < 0.05

The activities of TAT and PAL enzymes in *O. basilicum* leaves were significantly stimulated following treatments with conventional melatonin and MEL NPs (5, 10, and 20 µM) compared with the untreated control (Fig. 5). The obtained data revealed statisticaly significant differences among treatments (*P* < 0.05). The activity of TAT enzyme in untreated cotrol plant was 6.567 ± 0.035 U g^−^1 FW and PAL activity was 26.942 ± 0.234 U g^−^1 FW. Among the tested treatments, 10 µM MEL NPs elicitaed the most substantial response, significantly enhancing enzymatic activity. TAT activity incresed 20.429 ± 0.058 U g^−^1 FW (3.11-fold increase, *P* < 0.05). while PAL activity increased 41.938 ± 0.069 U g^−^1 FW (2.76-fold increase, *P* < 0.05) relative to the control. Furthermore, compared to conventional melatonin, 10 µM MEL NPs increased TAT and PAL activities by 1.51-fold and 1.55-fold, respectively, showing the superior efficacy of the nano-melatonin at this concentration by successfully stimulating important enzymes involved in phenylpropanoid production, suggesting a dose-dependent enhancement of metabolic activity. 30 µM MEL NPs showing significantly decreased the activity of TAT to 4.796 and PAL to 9.829 U g^−^1 FW, representing reductions of 0.730, 0.648 fold, respectively compared with the untreated control (*p* < 0.05) (Fig. 5).

### Gene expression

The transcription level of two selected genes, *4CL* and *HPPR* after 15 and 30 days of treatments, was analyzed using the 2⁻ΔΔCt method following treatments with MEL and MEL NPs of biological replicate (*n* = 3), with *Ubiquitin* as the reference gene. Expression levels are presented as fold change relative to the control group. Significant differences between treatments were found by statistical analysis (*P* < 0.05). *O. basilicum* leaves treated with 100 µ MEL moderate increases the expression of 4*CL* (1.223 ± 0.49- fold) after 15 and (1.507 ± 0.3-fold) after 30 days relative to the control. A similar pattern was observed for *HPPR* expression, which was significantly upregulated (1.831 ± 0.55-fold), (2.639 ± 0.05-fold) after 15 and 30 days, respectively compared to the untreated control. Treatment of MEL NPs at 5, 20 µM showed increasing in the 4*CL* gene expression level by 1.580 ± 0.71 and 2.763 ± 0.21-fold, respectively, as compared to the untreated control. The transcription levels of the *HPPR* gene were 2.054 ± 1.00 and 2.144 ± 0.26-fold in *O. basilicum* leaves treated with 5 and 20 µM MEL NPs at 15th day compared to the untreated plant, respectively.

**Fig. 6:**
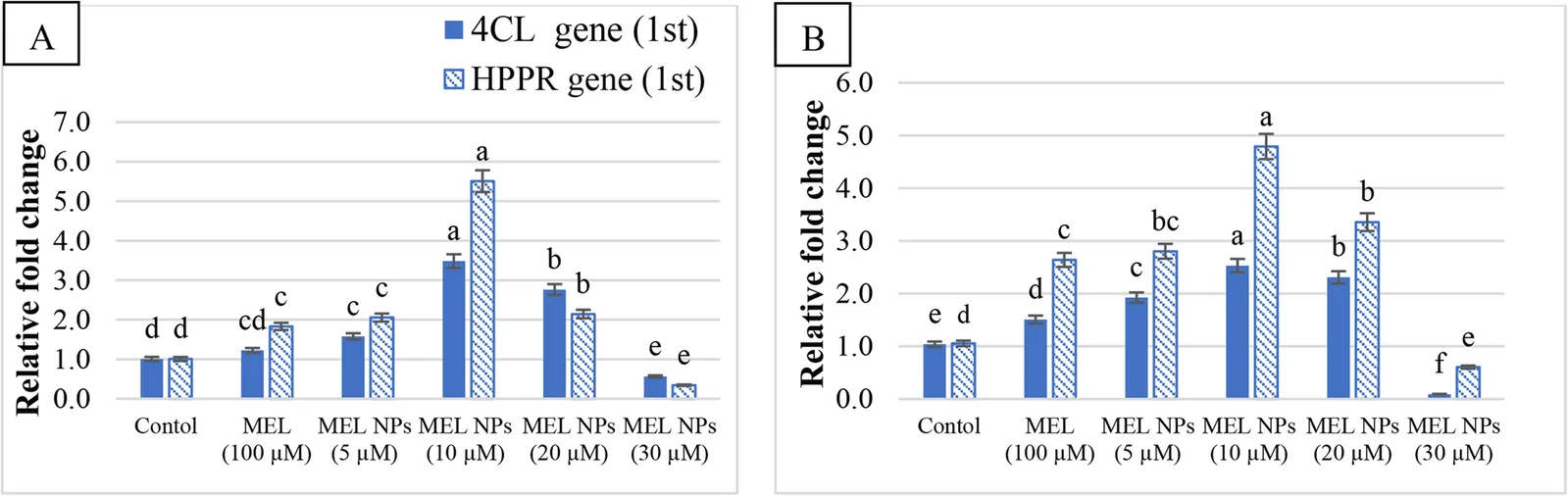
Relative fold changes in 4*CL* and *HPPR* gene expression levels determined by qRT-PCR in *Ocimum basilicum* L. leaves at 15 (**A**) and 30 (**B**) days post-treatment with control, conventional melatonin, and MEL NPs. Vertical bars represent the standard division (± SD) of technical replicates (*n* = 2). Different letters above the bars indicate significant differences using Duncan’s multiple range tests at *P* < 0.05

The greatest upregulation of both genes was observed in basil plants treated with 10 µM MEL NPs after 15 and 30 days (3.482 ± 0.40), (2.531 ± 0.80) for 4*CL* and (5.505 ± 1.03), (4.790 ± 0.79) for *HPPR*, respectively as shown in (Fig. 6). However, the transcription level of *4CL* and *HPPR* gene expression were downregulated 0.563 ± 0.07 and 0.343 ± 0.31- fold in plants treated with the highest nanoparticle concentration 30 µM, respectively.

## Discussions

At present, Nanoparticles have emerged as powerful tools in modern plant biotechnology due to their unique physicochemical properties and their ability to interact with biological systems at the molecular level. Their nanoscale size, high surface area, and enhanced reactivity allow them to influence various physiological and biochemical processes within plants [62]. In the context of secondary metabolite production, nanoparticles are increasingly recognized as effective elicitors capable of activating metabolic pathways related to plant defense and stress responses. When applied in controlled systems such as plant tissue culture, they can stimulate signaling cascades, modulate gene expression, and generate mild oxidative stress, all of which contribute to enhanced biosynthesis of valuable secondary metabolites [63]. These characteristics position nanoparticles as promising agents for improving the yield and quality of plant-derived compounds with significant pharmaceutical, nutritional, and industrial applications, highlighting their potential role in sustainable biotechnological practices [64].

The characterization of MEL NPs such as size, dispersion, shape, chemical composition, and surface charge was necessary to understand. Many factors affected the size and distribution such as molecular weight of MEL and CS, MEL/CS ratio, concentration of both, pH, temperature, and agitation rate [65]. The zeta potential indicated significant stability dispersions through electrostatic repulsion or attraction of positively charged nanoparticles. When an electrical double layer is present on the surface of colloidal particles which reduces the electrostatic repulsion between particles and precipitates, colloidal dispersion is stable [66, 67]. The positive net charge of the zeta potential supports the fact that the NPs have more strongly cationic particles to electrostatic interact with negatively charged components of biological membranes, thereby enhancing their intracellular uptake [68]. The difference in average diameter of prepared nanoparticles between DLS and TEM analysis may be due to the conditions of measurement involved. The high value of EE % indicated the entrapment of MEL into CS carrier through electrostatic interactions.

The FTIR data revealed vital information about functional groups to confirm the incorporation of MEL in CS nanoparticles as shown in Fig. 2. Melatonin was shown to peak at 1180 cm-1, which is consistent with the C–O stretching vibrations [69]. Also, the peak at 1492 cm^–1^ is sharper and may be due to aromatic –C = stretching vibration [46]. A peak is observed at 1550 cm^–1^ due to amide C = O bending vibration (amide II) [70]. The carbonyl group (C═O) is attributed to the absorption band at 1680 cm^−1^and the band at 3100 cm^–1^ is assigned to CH_2_ bending vibration [69]. who performed FTIR spectrum for melatonin and found that the C = O stretching vibration observed at 1681 cm^− 1^ and asymmetric CH_2_ stretching vibrations are generally observed in the region 3100 –3000 cm^− 1^ that agree with our results. Melatonin displayed a distinct peak for C-N bending at 3260 cm^− 1^ and a peak for N-H stretching at 3306 cm^− 1^. Our result agrees with the results obtained by [67]. The FTIR spectrum obtained for chitosan has a strong absorption band at 1080 cm^–1^, which is assigned to C − O stretching vibration. The band at 1370 cm^− 1^ is due to C − H bending vibration and at 1580 cm^− 1^ corresponds to the bending vibration of N − H. A peak is observed at 2870 cm^–1^ due to C − H stretching vibration. This result agrees with those obtained by [71]. The broad band at 3360–3450 cm^–1^ is associated with OH group stretching vibrations. This is in accordance with those obtained by [67], who found that the absorption band at 3437 cm^− 1^ corresponds to O–H group related to pure CS. There is a significant difference that can be observed between the FTIR spectrum obtained from the conventional MEL and chitosan and those obtained from melatonin-chitosan nanoparticles. The FTIR spectrum obtained for MEL NPs has a strong and broad absorption band at 3450 –3100 cm^–1^ corresponding to N − H stretching vibration overlapped with − OH stretching vibration. The broader peak indicated that hydrogen bonding has been enhanced. It agrees with the results obtained by [72] who found that the O-H axial bond stretching vibration and NH stretch are represented by the absorption band between 3440 and 3480 cm^− 1^.It is observed that the band at 1650 cm^− 1^ indicated amide (-CONH_2_) asymmetric stretching vibration, indicating the interaction between COO^−^ groups of CS and of NH groups of MEL. The appearance of intense bands was also noted at about 888, 1130, 1210, and 1530 cm^− 1^ corresponding to P − O−P and O − P = O, P = O, and N − O−P due to the interaction between melatonin, chitosan, and sodium tripolyphosphate. This result agrees with the results obtained b*y* [46].

MEL and MEL NPs have significant effects on TPC and TFC in basil plants which corrobotares the hypothesis that these compounds enhance antioxidant accumulation. phenolics and flavonoids content in basil leaves treated with 10 µM MEL NPS had significantly higher levels of 7.94 ± 0.114 mg g^− 1^ FW and 4.29 ± 0.211 mg g^− 1^ FW, respectively (Fig. 3). This finding align with earlier studies [73], which found that Melatonin boosted flavonoid production and activated the antioxidant capacity, delaying the aging of kiwifruit leaves. There is a linear correlation between the antioxidant activity and the phenolic content in *E. elatior* extract [74] due to their ability to reduce free radicals.The enhancement of these metabolites in *O. basilicum* leaves could be due to MEL inducing their biosynthesis. The augmentation of secondary metabolites in *O. basilicum* leaves is likely driven by melatonin-induced upregulation of their biosynthetic pathways. Specifically, the pronounced accumulation was observed in the 10 µM MEL-NPs treatment group compared to the untreated control, underscores the significant impact of this formulation on the physiological regulatory mechanisms governing phenolic and flavonoid synthesis. Furthermore, the intermediate effects recorded at concentrations of 5 µM and 20 µM MEL NPs clearly demonstrate a dose-dependent response, indicating that 10 µM is a physiological optimum where signaling pathways are optimally stimulated while maintaining cellular homeostasis, leading to improved molecular and biochemical responses. MEL may change the activity of important genes and related enzymes involved in phenolic metabolism [73, 75, 76]. Based on [18], melatonin enhances phenolic compound production by altering the NO and SA production and antioxidant enzymes activity. Also, MEL protects plant cells from environmental stress, such as oxidative stress, leading to increased production of phenols and flavonoids which have antioxidant properties [77]. The stimulatory effect of melatonin on phenolic content is ascribed to its capacity to initiate several metabolic pathways, thereby enhancing the biosynthesis of various phenolic compounds, particularly under stress conditions [78]. Our findings agreeing with the previous studies have reported that exogenous melatonin application increases phenolic accumulation across diverse plant species [79–81]. Similar results were attained by [82] who investigated that melatonin at a concentration of 100 µM displayed a maximum accumulation of phenol and flavonoid in *O. basilicum* L. Callus. The combination of 2,4-D (0.5 mg/L +kinetin (2 mg/L) + Fe₃O₄-NPs (4 mg/L)] significantly enhanced hypericin content by 66.6% as well as phenolic and flavonoid content in *Hypericum perforatum in vitro* culture according to [83]. The foliar application of the combination of nano silica and melatonin enhanced the total phenolic compounds in pea plant [84]. In a previus study, the content of phenols and flavonoids enhanced because of the synergy of a nitrophenol-based biostimulant nanocomplex in tomatoes [85].

Phenolic productions are directly proportional and strongly dependent on the activation of key enzymes (tyrosine ammonia lyase) responsible for Phyto-chemical production [86]. In addition, Melatonin’s recognized function to boost plants’ antioxidant systems may have led to an increase in flavonoid synthesis [27]. This action is probably mediated through transcriptional regulation, as melatonin has been demonstrated to modify gene expressions associated with flavonoid synthesis pathways [46]. The activation of key enzymes such as phenylalanine ammonia-lyase (PAL) and chalcone synthase (CHS) may have increased flavonoid production. Additionally, the increased accumulation of flavonoids may have been caused by the complex hormonal interactions between melatonin and other plant hormones, such as salicylic acid and jasmonic acid, which are involved in secondary metabolite formation and stress signaling [87]. Furthermore, it has been demonstrated that chitosan stimulates defense responses in plants, including the production of secondary metabolites. It is possible that the chitosan component of the nanocarrier system performed in concert to produce these metabolites [48]. It is possible that the chitosan and melatonin combination in nano formulation enhanced flavonoid production in a synergistic manner.

The synthesis of important secondary metabolites in *Ocimum basilicum* tissues were significantly enhanced by MEL NPs particularly at 10 µM, showing that rosmarinic acid was the most abundant content (19.34 mg. g-^1^ DW) (Fig. 4), which is the highest potent therapeutic compound. The MEL NPs at this dose most likely offered the best cellular absorption and sustained release, guaranteeing adequate intracellular availability without inducing toxicity or metabolic imbalance. These findings are consistent with previous research on the influence of melatonin in plant stress responses and secondary metabolite synthesis [83, 87]. The precise mechanism by which melatonin or nano-melatonin induces rosmarinic acid production remains incompletely elucidated. However, several potential pathways have been proposed: (a) melatonin functions as a potent antioxidant, effectively scavenging reactive oxygen and nitrogen species, thereby mitigating oxidative stress [21, 88, 89]; (b) melatonin regulates the expression of genes and modifies the activity of key enzymes involved in the biosynthetic pathway of rosmarinic acid [16]. Similarly, application of 100 µM melatonin increased rosmarinic acid content by about 75% in *Salvia officinalis*, with a subsequent decline observed at 200 µM [90]. Likewise [16], demonstrated that 100 µM melatonin enhanced rosmarinic acid accumulation in *Dracocephalum kotschyi* leaves under salinity stress. Additionally [9], reported a nearly fivefold increase in rosmarinic acid levels in *Ocimum basilicum* callus treated with 100 µM melatonin compared to controls.

The biosynthesis proceeds with the independent transformation of the aromatic amino acids L-phenylalanine and L-tyrosine [91]. The enzymes involved in RA synthesis as well as the biosynthetic pathways beginning with both amino acids. The results depicted in Fig. 5 reveal a notable increase in TAT and PAL activity of basil plant tissue treated with conventional and nanoform. The most significant increase was in tissues treated with MEL NPs 10 µM showing activity of 20.429 ± 0.058 U g^−^1 FW and 41.938 ± 0.069 U g^−^1 FW (*P* < 0.05), respectively compared to untreated control. These observations correlate with previous studies on the role of melatonin in boosting the activity and the proportion of defensive enzymes, including CAT, POD, SOD, β-1,3-glucanase, chitinase (CHI), phenylalanine ammonia lyase (PAL), and polyphenol oxidase (PPO) [92]. The activity of TAT and PAL were sign indicated that melatonin contributes to enhanced nutrient uptake and transport is essential for enzymes function, thereby indirectly stimulating enzyme activity as reported by [93] who stated that exogenous application of melatonin regulates calcium influx and modulates the expression of calmodulin-related genes. Elevated intracellular calcium activates various enzymes, including PAL which is a key catalyst in rosmarinic acid biosynthesis. Calcium also plays a crucial role in regulating gene expression of enzymes involved in phenolic acid production. Our results are in agreement with [16] who observed increased TAT and PAL activities in *Dracocephalum kotschyi* treated with 100 µM melatonin under salinity stress. Similar enhancements in enzyme activity have been reported in *pomegranate* [94] and tomato fruits [95, 96]. also demonstrated melatonin-induced stimulation of phenylpropanoid pathway enzymes, including PAL. Moreover [18], documented a dose-dependent increase in PAL activity with melatonin treatment, although no significant effect on TAT activity was observed.

According to this study, the application of nano-melatonin (MEL NPs) significantly altered the biosynthesis of rosmarinic acid in *O. basilicum* by means of coordinated regulation at the transcriptional, enzymatic, and metabolic levels. The phenylpropanoid pathway is one of the most important metabolic networks in plants and responsible for producing an extensive variety of aromatic compounds that support ecological interactions, stress tolerance, structural integrity, and specialized secondary metabolism [25]. Beginning with deamination of phenylalanine, this pathway produced several enzymes that are essential intermediates for the synthesis of lignin, flavonoids, and various phenolic derivatives [97]. Additionally, the NPs stimulated the synthesis of both primary and specialized metabolites by alerting the gene expression of key enzymes in metabolic pathways [98, 99]. To reveal the detailed regulatory mechanisms underlying RA synthesis in basil, we used correlation analysis to establish the gene-to-metabolite network under MEL NPs treatment where there is a close relationship between genes and desired metabolites. Biogenetic and molecular research on *Mentha* and *Coleus blumei* suggests a biosynthetic pathway where in 3,4-hydroxyphenyl lactic acid and 4-Cumaryl-CoA esters are used to construct rosmarinic acid by regulating the expression of *C4H*, *HPPR*, and *RAS* genes. In their evaluation of transcriptome alterations in six *Dracocephalum tanguticum* tissues [100], found that 22 genes—including two *HPPR* genes, five *RAS* genes, three *C4H* genes, five 4*CL* genes, three *TAT* genes, and four *PAL* genes—were involved in the manufacture of rosmarinic acid. Within this metabolic network, *4CL* and *HPPR* were screened as key candidate genes due to their strong correlation with rosmarinic acid (RA) accumulation, representing the phenylalanine-derived and tyrosine-derived branches, respectively, of the RA biosynthetic pathway [101]. The present results revealed that the transcription level of 4*CL* and *HPPR* gene expression were down-regulated in the plant treated with Nano-melatonin at concentration of 30 µM Fig. 6, while the level of these gene expressions was up-regulated in the plants treated with normal melatonin and Nano-melatonin at 5, 10, 20 µM compared to untreated control Fig. 6. The overexpression levels were observed in the plant treated with 10 µM Nano-melatonin. This effect is attributed to melatonin’s role in upregulating specific genes essential for the stimulation of rosmarinic acid biosynthesis. Consistent with our results [16], reported that MEL at 100 µM upregulated the expiration of PAL and RAS gene in salt stressed leaves of *Dracocephalum kotschyi* [96]. stated that *Gastrodia elata* treated with MEL enhanced the expression of phenylalanine ammonia-lyase (PAL), cinnamate-4-hydroxylase and 4-coumarate: CoA ligase (4*CL*) [95]. reported that melatonin treatment significantly upregulated *PAL* gene expression, leading to enhanced phenolic accumulation in tomato fruit. The superior performance of melatonin-loaded chitosan nanoparticles (MEL-CSNPs) relative to free melatonin suggests that nano-encapsulation confers distinct physiological advantages [102]. This enhanced efficacy is likely attributable to the sustained-release profile of the nano-formulation, which ensures a continuous supply of melatonin to support plant physiological functions over an extended period. Furthermore, the nano-composition likely facilitates improved systemic absorption and cellular dispersion within plant tissues [103]. Collectively, these mechanisms-controlled release and enhanced bioavailability explain the heightened capacity of MEL-NPs to stimulate rosmarinic acid production compared to conventional melatonin treatments.

The results indicate that the efficacy of melatonin is strictly dose-dependent, reflecting a specific physiological sensitivity in *O. basilicum*. Our findings demonstrate that 10 µM MEL NPs represents a critical threshold concentration for achieving peak metabolic enhancement. At lower concentrations, the signaling strength might not be strong enough to activate important genes and enzymes involved in the synthesis of phenylpropanoid. The observed decline in effectiveness at the higher dosage (30 µM) suggests a biphasic response, where exceeding the optimal concentration may lead to diminishing returns or potential phytotoxicity. These results align with the broader understanding of melatonin’s pleiotropic functions in plant physiology [104]; it acts not only as a potent antioxidant but also as a signaling molecule that orchestrates gene expression and various metabolic pathways [12].

While this work showed how MEL NPs can significantly increase plant secondary metabolism, there are a number of limitations that need to be noted. The in vitro nature of the study may not fully capture the physiological complexity of whole plants grown in soil, where rhizosphere interactions and environmental stressors play a critical role in determining plant responses. Moreover, the possibility of dose-dependent phytotoxicity at higher concentrations, together with the absence of direct quantification of nanoparticle uptake and intracellular stability. Because the study was limited to a single basil genotype, the results may not be easily extrapolated to other cultivars with varying degrees of metabolic plasticity. Lastly, Chitosan/TPP without melatonin, the nanoparticle control, was not included. Therefore, as it is impossible to completely rule out the possibility of chitosan nanoparticles’ inherent bioactivity, chitosan carrier functions as a bioactive elicitor that independently modifies phenolic metabolism, the observed reactions cannot be solely ascribed to melatonin nano-delivery. These factors suggest that the observed results are due to the carrier’s synergistic effect rather than the unique function of the encapsulated melatonin. However, the main goal of this investigation was not to examine the separate biological effects of the chitosan carrier, but rather to evaluate the effectiveness of melatonin nano-encapsulation.

These achievements in our research could lead to pharmaceutical applications through the standardized, large-scale production of plant-derived antioxidants and bioactive compounds. Consequently, future follow-up experiments should transition to soil-grown plants to assess efficacy under invitro-like conditions and examine the kinetics of melatonin release for ensuring sustained delivery. From a pragmatic standpoint, creating affordable synthesis methods will be crucial to widespread application. more comprehensive understanding of nano-priming would come from investigating more extensive secondary metabolite pathways, such as terpenoids and alkaloids.

## Conclusion

The current study was designed to evaluate the use of nanotechnology and its recent applications development. The results indicated that melatonin loaded chitosan nanoparticles, particularly the concentration of 10 µM was the most effective concentration enhancing the biosynthesis of rosmarinic acid in *Ocimum basilicum* by modulating key enzymatic activities (TAT and PAL) and upregulating the expression of pivotal biosynthetic genes (4*CL* and *HPPR*) and significantly increasing phenolic and flavonoid contents, outperforming the effects of traditional melatonin treatments under *invitro* conditions. These findings show that nano-delivery increases melatonin’s bioavailability and cellular uptake, increasing metabolic flux via tyrosine-derived and phenylpropanoid pathways. By coordinating the transcriptional and enzymatic regulation of important biosynthetic pathways and nano-melatonin offers a promising approach to increase the production of rosmarinic acid metabolites in basil plants.These results suggest that the plant hormone loaded NPs may be a useful tool for optimizing the metabolic profiles of the plants for biotechnological applications. However, more research is necessary to confirm these results in soil-grown plants, investigate the uptake and stability of nanoparticles, and assess responses across various genotypes. Further exploration of the underlying molecular pathways may unlock innovative strategies for sustainable production of high-value phytochemicals in agricultural and pharmaceutical applications.

## Data Availability

All research data supporting the results and analysis of the article could be shared upon request.

## Abbreviations

NPs: Nanoparticles
MEL: Melatonin
CS: Chitosan
TPC: Total phenol content
TFC: Total flavonoids content
TAT: Tyrosine aminotransferase
PAL: Phenylalanine ammonia-lyase
*HPPR*: Hydroxyphenylpyruvate reductase
4*CL*: 4-Coumarate: CoA ligase
TEM: Transmission electron microscopy
DLS: Dynamic light scattering
FTIR: Fourier transform infrared spectroscopy
